# P-364. Outbreak of *bla-*OXA-48 in the eastern part of Singapore

**DOI:** 10.1093/ofid/ofae631.565

**Published:** 2025-01-29

**Authors:** Edwin Conceicao, Hau Yiang Cheong, Thean Yen Tan, Li Jie, Suhailah Binte Nasir, Toudi Wu, Maria Theresa, Crystal Wong, Ruby Foo, Seow Yen Tan

**Affiliations:** Changi General Hospital, Singapore, Not Applicable, Singapore; Changi General Hospital, Singapore, Not Applicable, Singapore; Changi General Hospital, Singapore, Not Applicable, Singapore; Changi General Hospital, Singapore, Not Applicable, Singapore; Changi General Hospital, Singapore, Not Applicable, Singapore; Changi General hospital, Singapore, Not Applicable, Singapore; Changi General Hospital, Singapore, Not Applicable, Singapore; Changi General Hospital, Singapore, Not Applicable, Singapore; Changi General Hospital, Singapore, Not Applicable, Singapore; Changi General Hospital, Singapore, Not Applicable, Singapore

## Abstract

**Background:**

Changi General hospital (CGH) is a 1000 bed acute hospital that mainly serves the general population in the eastern region of Singapore and community. At the beginning of October 2023 till March 2024, we observed an increase in the number of the Carbapenemase producing *Enterobacteriaceae* (CP-CRE) *bla-*OXA-48 cases from the routine long-stayer inpatient surveillance screening in several wards.

**Methods:**

Standard outbreak protocols are executed, including enhanced environmental testing and disinfection, inpatient contact tracing, and Box-PCR typing of *Klebsiella pneumoniae spp*. isolates.

**Results:**

68% (n = 226) of the *bla-*OXA-48 are identified from the outbreak wards, of which 15% (n = 33) cases is likely due to the additional beds added in the outbreak wards, reported an OR of 3.6 times (*p*-value: 0.007, 95%CI: 1.3 – 9.0) of being colonized by *bla-*OXA-48 CP-CRE compared to the non-outbreak wards. Patients from the outbreak wards have 2.7 times higher odds of requiring significantly more nursing care compared to the non-outbreak wards (*p*-value: 0.009, 95%CI: 1.3 – 5.7). The limited space to manoeuvre during nursing care, especially during diaper changing for the bed bound patients which is 61% (n = 139) of *bla-*OXA-48 CP-CRE cases in the outbreak wards. Among the bedbound patients, the odds of being colonized with *bla-*OXA-48 CP-CRE is 5.6 times (*p*-value: 0.008, 95%CI: 1.3 – 24.7) in the outbreak wards compared to the non-outbreak wards due to possible surface contamination when the curtains are drawn during the diaper changing or through accidental contact with the adjacent beds by the healthcare workers.

The microbiological typing suggests 2 common types are circulating between patients in the outbreak wards in 79% (n = 58) of the samples tested. 3% (n = 194) of the environmental samples are positive for *bla-*OXA-48 detected in the toilet and drains, used only by ambulating patients during their hospitalization. No incidences (n = 75) of community onset of *bla-*OXA-48 CP-CRE is reported form a point prevalence survey (PPS).

**Conclusion:**

Hence, *bla-*OXA-48 CP-CRE transmission is more likely from healthcare worker transmission. Ensuring adequate spacing between hospital beds is crucial to facilitate the work of healthcare staff, allowing them to move freely and efficiently while providing care.

**Disclosures:**

**All Authors**: No reported disclosures

